# Update on Cannabidiol Clinical Toxicity and Adverse Effects: A Systematic Review

**DOI:** 10.2174/1570159X21666230322143401

**Published:** 2023-09-01

**Authors:** Graziella Madeo, Ashita Kapoor, Raffaele Giorgetti, Francesco Paolo Busardò, Jeremy Carlier

**Affiliations:** 1 Clinical Center of Neurology and Psychiatry, Brain&Care Group, Rimini, Italy;; 2 Unit of Forensic Toxicology, Section of Legal Medicine, Department of Biomedical Sciences and Public Health, Marche Polytechnic University, Ancona, Italy

**Keywords:** Cannabidiol, clinical study, toxicity, adverse event, psychosis, anxiety, treatment-resistant epilepsy

## Abstract

**Background:**

Compelling evidence from preclinical and clinical studies supports the therapeutic role of cannabidiol (CBD) in several medical disorders. We reviewed the scientific evidence on CBD-related toxicity and adverse events (AEs) in 2019, at the beginning of the spike in clinical studies involving CBD. However, CBD safety remained uncertain.

**Objective:**

With the benefit of hindsight, we aimed to provide an update on CBD-related toxicity and AEs in humans.

**Methods:**

A systematic literature search was conducted following PRISMA guidelines. PubMed, Cochrane, and Embase were accessed in October 2022 to identify clinical studies mentioning CBD-related toxicity/AEs from February 2019 to September 2022. Study design, population characteristics, CBD doses, treatment duration, co-medications, and AEs were compiled.

**Results:**

A total of 51 reports were included. Most studies investigated CBD efficacy and safety in neurological conditions, such as treatment-resistant epilepsies, although a growing number of studies are focusing on specific psychopathological conditions, such as substance use disorders, chronic psychosis, and anxiety. Most studies report mild or moderate severity of AEs. The most common AEs are diarrhea, somnolence, sedation, and upper respiratory disturbances. Few serious AEs have been reported, especially when CBD is co-administered with other classes of drugs, such as clobazam and valproate.

**Conclusion:**

Clinical data suggest that CBD is well tolerated and associated with few serious AEs at therapeutic doses both in children and adults. However, interactions with other medications should be monitored carefully. Additional data are needed to investigate CBD's long-term efficacy and safety, and CBD use in medical conditions other than epilepsy syndromes.

## 1 INTRODUCTION

 Cannabidiol (CBD) is a prevalent bioactive compound in the Cannabis sativa plant, second to Δ9-tetrahydrocannabinol (THC) but without eliciting psychoactive effects [1-4]. CBD's precise molecular mechanism is still not fully elucidated. Compared to THC, CBD shows a very low affinity for endogenous cannabinoid receptors CB1 and CB2 [5] and actually seems to antagonize the CB1 receptor by allosteri CBD is the first cannabis-based medicine approved by both the US Food and Drug Administration (FDA) and the European Medicines Agency (EMA) as an add-on treatment for two rare and severe forms of childhood epilepsies: Lennox-Gastaut Syndrome (LGS) and Dravet Syndrome (DS) [13]. Recent clinical trials have also investigated CBD as a potential treatment for diabetes [14], fatty liver disease [15], insomnia [16, 17], psychosis [18, 19], autism [20, 21], chronic pain [22], cancer [23], and neurodegenerative disorders, such as Parkinson’s disease (PD) and Alzheimer’s disease (AD) [24-26]. CBD is also available in over-the-counter preparations that have not been evaluated in clinical trials [27].

Considering the recent approval by the FDA and EMA for childhood epilepsy syndrome and the wide range of pharmacological effects with a broad spectrum of potential clinical use, CBD safety needs to be investigated. In 2019, we reviewed the scientific evidence on CBD-related toxicity and adverse events (AEs) [28]. However, most clinical studies involving CBD were published in recent years, providing essential data on CBD safety at specific controlled doses in humans. The aim of the present review was to provide an update on CBD toxicity and AEs, specifically focusing on clinical trials.

## 2 MATERIALS AND METHODS

A systematic literature search was conducted according to the PRISMA guidelines [29]. Report identification, screening, and assessment for eligibility and inclusion were conducted by two authors independently after agreeing on the search term list. The results were cross-checked after each step of the investigation.

### 2.1 Report Identification

PubMed (US National Library of Medicine), Cochrane, and Embase bibliographic databases were accessed in October 2022 to identify scientific reports of CBD-related toxicity and AEs. Other potential reports were searched from international agencies or institutional websites including ClinicalTrials.gov and US FDA. The search terms were: “cannabidiol”, “CBD”, or “Epidiolex”; in combination with “adverse effects”, “side effects”, “adverse reactions”, “adverse events”, “toxicology”, “toxicity”, or “safety”. Further manuscripts were retrieved through the reference lists of selected articles. Duplicates were excluded based on the articles’ DOI using Microsoft Excel (v. 2211); the results were manually checked before removal. Only the studies published after February 2019 were included to avoid redundancy with our previously published article [28].

### 2.2 Report Screening and Assessment for Eligibility

From the list of identified reports, clinical studies with a title and abstract mentioning CBD use were eligible for full-text reading. If a title/abstract was insufficient to understand whether the corresponding report should be included, the report was eligible for full-text reading. Clinical studies on CBD with THC co-administration only and case reports were excluded, as observed adverse events and toxicity can hardly be linked to CBD use. Literature reviews, commentaries, letters to the editor, viewpoints, or erratum; purely analytical (method development and chemical analysis), *in vitro* or preclinical studies; and reports that were not written in English, Italian, or French were excluded.

### 2.3 Report Inclusion

The full text of manuscripts reporting CBD toxicity or AEs in clinical studies was included. Clinical studies on CBD with THC co-administration; case reports; literature reviews, commentaries, letters to the editor, viewpoints, or erratum; purely analytical, *in vitro*, or preclinical studies; and reports that were not written in English, Italian, or French were excluded, if not excluded during the report identification and screening steps.

Authorship, date of publication, study title, study design, population characteristics, CBD doses, treatment duration, co-medications, and AEs were compiled.

The studies were compared based on their location and date of occurrence to avoid duplicates. If it arose, data were merged into a single entry in the database. The authors’ affiliations, funding sources, and disclosures were verified for each report to avoid potential bias linked to conflicts of interest.

## 3 RESULTS

**Fig. (1):**
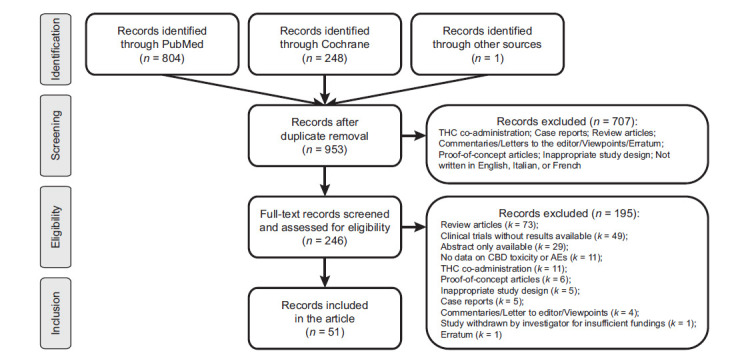
Flow diagram of the literature search.

The results of the systematic review are presented in a flow diagram of the literature search (Fig. **1**). The initial search strategy yielded a total of 1053 articles that were screened by title and abstract for eligibility. One hundred duplicates were removed. Of the 953 remaining records, 707 were removed as they included preclinical studies, inappropriate study design/population, and THC co-administration. A total of 246 studies were considered for full-text review. Forty-nine reports referring to clinical trials in a recruiting, not yet recruiting, or completed status with no results posted, and 29 abstracts were excluded because of missing results or not enough data on population characteristics and CBD toxicity. Likewise, six proof-of-concept papers and five case reports were excluded as they did not meet the inclusion criteria. Finally, a total of 51 reports met the inclusion criteria of this systematic review (Table **1**). Study design, population characteristics, CBD doses, treatment duration, co-medications, and AEs are depicted in Table **1**. The database compiling all the retrieved data is available on demand.

### 3.1 Neurological Studies

Since the FDA and EMA approval of CBD for childhood epileptic syndrome, there has been an increase in clinical studies focusing on the long-term efficacy and safety of CBD, and its use in several forms of treatment-resistant epilepsies (TREs). Of 51 included reports, 24 studies evaluated CBD use as an add-on treatment for severe TREs, including LGS, DS, Tuberous Sclerosis complex (TSC), and other resistant epileptic syndromes. The study designs included: open-label trials [30-39], observational cohort studies “open-label extension” (OLE) [40-43], retrospective chart reviews [44, 45], and randomized controlled trials (RCTs) with placebo group comparison [46-51]. Most studies aimed to assess the efficacy and safety of CBD treatment, and study endpoints included seizure frequency reduction, seizure response rate, seizure freedom, change in seizure severity, treatment discontinuation, and occurrence of AEs.

In the OLE studies, LGS, DS, and TSC patients who completed the RCTs were enrolled in a long-term open-label extension trial for each pivotal study allowing the evaluation of treatment-emergent AEs related to CBD administration. Across the trials, a pharmaceutical formulation of highly purified CBD (Epidiolex; 100 mg/mL) in oral solution was administered as an add-on treatment to the pre-existing antiepileptic regimen at up to 50 mg/kg/day with a follow-up time of 48 weeks. Most patients reported at least one AE, whose severity was mostly mild or moderate. The most commonly reported AEs in patients with LGS, DS, and TSC were pyrexia and gastroenteric tract disorders, including diarrhea, vomiting, and decreased appetite [40, 41, 43, 47, 52]. Somnolence, sedation, drowsiness, and convulsion were the most frequent neurological AEs. Overall, the reported AEs, frequencies, and severity, are comparable with previous observations in the RCTs [46, 51]. Treatment discontinuation was associated with increased liver transaminase and seizures. Elevation of liver transaminase was more frequent in patients taking concomitant valproate and higher dosages of CBD.

An open-label, multicenter, expanded access trial reported similar findings in children with TRE, ineligible to be included in RCTs that only included patients with LGS or DS [42]. All 47 enrolled patients experienced at least one AE during the 36-month treatment. Most AEs were mild or moderate in severity. Serious AEs were reported by twelve children; none of them were related to CBD according to the authors. The most common AEs were upper respiratory infections, gastrointestinal disorders (diarrhea, nausea, vomiting), pyrexia, and somnolence [42]. A well-tolerated safety profile of oral CBD and a good response rate for controlling seizures was confirmed by an expanded access program trial involving a large cohort of 607 patients (58 with DS, 94 with LGS, and 455 with other TREs) over a 96-week treatment [31]. Patients received oral CBD titrated until tolerability to a maximum dose of 25-50 mg/kg/day and were taking a median of three concomitant antiepileptic drugs (AEDs) (range 0-10). After 144 weeks, the safety analysis revealed that 91% of patients reported at least one AE. Most AEs were mild or moderate in severity; diarrhea and somnolence were the most common (30% and 24%, respectively).

A good antiseizure response to CBD, defined as ≥ 50% reduction in seizure frequency, and an acceptable safety profile have been demonstrated by studies evaluating both children and adults with other forms of TREs [32, 45], such as developmental and epileptic encephalopathy [37, 38, 53], refractory childhood-onset epileptic spasm [34], X-fragile syndrome [54], and *SYNGAP1* developmental and epileptic encephalopathy [55]. Most patients were taking a highly purified oral CBD formulation; in only one study, a transdermal CBD gel at a dosage range of 150-500 mg/day was administered [37]. Most AEs were mild or moderate. The most common AEs were related to gastrointestinal (diarrhea, nausea, decreased appetite), and respiratory or neurological disturbances (somnolence and sedation) [37]. Overall, a few serious AEs, including increased transaminase level, sedation, and convulsion, were associated with the discontinuation of treatment.

Considering the increasing interest in CBD therapy for epilepsies, concerns regarding potential cognitive effects have been raised by treating physicians. Three open-label trials assessed whether CBD has long-term cognitive effects in both children and adults with TRE [30, 33, 36]. 27 adults and 38 subjects between the age of 3 and 19 years with TRE enrolled in open-label CBD studies were evaluated prior to initiating CBD treatment and after one year. There were no statistically significant changes in cognitive functions, as measured by the National Institutes of Health Toolbox Cognition Battery, or functional adaptive skills, as measured by the Adaptive Behavior Assessment System - Second edition after a one-year trial with CBD [33, 36]. Similarly, no significant CBD-related behavioral effects were found in 39 patients with TRE, including both children and adults, evaluated at baseline and after three months of CBD treatment [30].

Finally, four studies were primarily aimed at describing pharmacokinetic analyses or DDI between CBD and antiseizure or non-antiseizure medications [44, 49, 50, 56]. An open-label, multiple-ascending-dose, phase I/II trial characterized the pharmacokinetic parameters and short-term tolerability of a pharmaceutical-grade synthetic CBD oral solution in 61 pediatric patients (aged 1 to ≤ 17 years) with TRE as an add-on to their AED regimen [56]. Patients received a single dose (5, 10, or 20 mg/kg) on day 1 and twice-daily dosing on days 4 through 10 (10-mg/kg (cohort 1), 20-mg/kg (cohort 2), or 40-mg/kg (cohort 3) total daily dose). A steady-state concentration of CBD was reached at 2-6 days of twice-daily dosing. Systemic CBD exposure generally increased linearly with increases in dose. A bidirectional DDI occurred with CBD and clobazam. Concomitant administration of clobazam with oral CBD at 40 mg/kg/day resulted in a 2.5-fold increase in mean CBD exposure. All doses were generally well tolerated, and common AEs in over 10% of patients were somnolence (21.3%), anemia (18.0%), and diarrhea (16.4%) [56].

A retrospective chart review study investigated a DDI between CBD and mTOR inhibitors, such as everolimus and sirolimus, in a cohort of 25 patients with TSC (18 with everolimus, 7 with sirolimus) [44]. The median age at treatment initiation was 17 years (range 3-45 years; 13 children, 12 adults). CBD was orally administered, and follow-up mTOR inhibitor levels were drawn after a therapeutic dose of 5-20 mg/kg/day was achieved. Both everolimus and sirolimus plasma levels were significantly higher in 76% of patients after CBD treatment. AEs occurred in 10 of 25 patients (40%) with diarrhea being the most frequent AEs in three patients (12%). Other AEs included drowsiness, increased and severe mouth sores, increased acne, ankle swelling, sinusitis, abdominal pain, mild elevation of transaminases, and increased phenytoin level [44].

A phase II, two-arm, parallel-group, double-blind, randomized, placebo-controlled trial was conducted to evaluate pharmacokinetic DDI between CBD and stiripentol or valproate in 35 patients with epilepsy aged 16-55 [50]. Patients receiving a stable dose of stiripentol or valproate were randomized 4:1 to receive concomitant double-blind CBD or placebo. Oral CBD was administered at a dose of 20 mg/kg/day from day 12 to 26, following a 10-day dose escalation. The combination of CBD and stiripentol led to a small increase in exposure to stiripentol, while the combination with valproate did not result in significant changes in the pharmacokinetics of valproate or its metabolite, 2-propyl-4-pentenoic acid (4-ene-VPA). The most common AE was diarrhea; most AEs were mild. Two patients discontinued CBD because of serious AEs (rash (n = 1) in the stiripentol arm; hypertransaminasemia (n = 1) in the valproate arm) [50].

A similar trial investigated potential DDIs between CBD and clobazam in 20 patients with refractory epilepsy aged 18-65 on stable clobazam doses [49]. No statistically significant pharmacokinetic changes were found in patients taking concomitant CBD and clobazam for 32 days. A well-tolerated safety profile for CBD was reported, with all AEs being mild (31.3%) or moderate (43.8%) in severity. Most common AEs were mild (5 (31.3%)) or moderate (7 (43.8%)). The most common AEs were diarrhea, nausea, vomiting, sedation, and somnolence. One severe AE (6.3%) occurred as a seizure cluster leading to withdrawal. There were two cases (12.5%) of increased transaminase level with concomitant valproate, and three cases (18.9%) required a reduction of CBD dose due to the occurrence of rash, diarrhea, and multiple events of sedation, slurred speech, and word-finding difficulties [49].

CBD is a modulator of the endogenous cannabinoid system, involved in the homeostatic regulation of many physiological processes, such as mood, anxiety, sleep, and neuroprotection, which seem to be involved in mechanisms underlying the pathophysiology of PD. A phase II/III, double-blind, placebo-controlled clinical trial was conducted in 33 PD patients with rapid eye movement behavior disorder. Patients were randomized 1:1 to CBD in doses of 75 to 300 mg or matched capsules placebo and were followed up for 14 weeks [24]. CBD showed no reduction of rapid eye movement behavior disorder manifestation in PD patients; AEs were similarly reported in both groups.

A transdermal CBD gel was also evaluated for efficacy and safety profile in a RCTs, double-blind, placebo-controlled, multicenter trial [48]. A total of 188 patients (mean age 39.2 (12.78)) with focal resistant epilepsy were randomized (1:1:1) to 195-mg or 390-mg transdermal CBD or placebo daily for 12 weeks, after which they could enroll in an OLE study for up to 2 years. AEs occurred in 50.4% of the CDB group *vs.* 41.3% of the placebo group at a similar rate [48].

### 3.2 Psychiatric Studies

In psychiatry, CBD has been suggested to have antipsychotic, antidepressant, anxiolytic, anti-craving, and precognitive effects [1-3]. Our systematic search has led to the inclusion of twelve clinical trials, of which five are RCTs, double-blind, placebo-controlled studies [57-61], six open-label trials [19, 62-64], and one retrospective case series [65].

CBD has been evaluated for its anxiolytic properties in an RCT, double-blind placebo-controlled, in which 80 patients (aged 18-65 years) with a treatment-refractory social anxiety disorder or panic disorder with agoraphobia were enrolled. Patients underwent eight therapist-assisted exposure *in vivo* sessions (weekly, outpatient). Prior to each session, patients were administered 300 mg oral CBD (n = 39) or placebo (n = 41). Although no differences were found in treatment outcome over time between CBD and placebo as measured by the Fear Questionnaire assessed at baseline, mid-, and post-treatment, and at 3- and 6-month follow-up, only a few AEs were reported, evenly distributed between the CBD (n = 4) and the placebo groups (n = 6). Most reported AEs were dizziness, drowsiness, recurrent tiredness, the recurrent feeling of a strong blood flow, and recurrent headache [58]. In an open-label study enrolling 31 young subjects aged 12-25 years with treatment-refractory anxiety disorder, anxiety severity scores significantly decreased after receiving CBD for 12 weeks at 400-800 mg. CBD demonstrated an acceptable safety profile with no serious AEs reported. Most patients (80.6%) experienced mild or moderate AEs. The most common AEs were fatigue, low mood, hot flashes, and cold chills [63]. A more recent open-label trial used CBD for the reduction of emotional an effective agent for the reduction of emotional exhaustion and burnout symptoms in frontline healthcare professionals working with patients with COVID-19. A total of 120 participants were randomized to receive either CBD, 300 mg, plus standard care (treatment arm; n = 61) or standard care alone (control arm; n = 59) for 28 days. Five participants who received CBD plus standard care experienced serious AEs, including increased liver transaminase levels and skin rash, with full recovery after discontinuation [66]. Most AEs were mild or moderate in severity (> 10%). Somnolence (n = 34 (28.8%)), fatigue (n = 27 (22.9%)), increased appetite (n = 19 (16.1%)), diarrhea (n = 13 (11.0%)), weight gain (n = 12 (10.2%)), lethargy (n = 12 (10.2%)) were frequently reported.

In a retrospective case series, data from 11 patients diagnosed with post-traumatic stress disorder were evaluated to investigate CBD efficacy and safety profile. CBD was given in a flexible dosing regimen in two different formulations, capsular and liquid spray (n = 4 only capsular; n = 1 only liquid spray; n = 6 both capsular and liquid spray). The mean total starting dose of CBD (liquid or capsular or both) was 33.18 mg (standard deviation = 23.34). The mean total dose of CBD at the conclusion of the study period was 48.64 mg (range 2-100). Patients were taking concomitant medications of different classes; the most relevant were AEDs, antidepressants, antipsychotics, anxiolytic/sedatives, and beta-blockers. CBD was well tolerated with no serious AEs reported and few mild/moderate AEs [65].

Likewise, CBD showed an acceptable safety profile in an infantile population with severe behavioral problems and intellectual disabilities in an RCT, double-blind, placebo-controlled trial conducted by Efron *et al*. [59]. Eight children aged 8-16 years receiving oral CBD at a maximum dose of 500 mg/day reported only a few mild/moderate AEs. No reduction adjustments due to AEs were necessary, and no withdrawals or serious AEs were recorded [59]. No significant serious AEs were also reported by studies evaluating oral CBD at a range dose of 200-800 mg for a duration of 3 to 28 days in a cohort of subjects with different types of addiction, such as cannabis use disorder [57], opioid use disorder [60, 64], cocaine use disorder [61], and tobacco use disorder comorbid to psychotic syndromes [19]. Across the studies, most AEs reported were mild or moderate in severity. Nausea, diarrhea, fatigue, tiredness, and headache were commonly reported.

### 3.3 Drug-Drug Interaction and Pharmacokinetic Studies

CBD is metabolized in the liver and the intestine by cytochrome P450 (CYP) CYP2C19 and CYP3A4, and 5'-diphosphoglucuronosyltransferase (UGT) UGT1A7, UGT1A9, and UGT2B7 isoforms, mainly producing hydroxylated and carboxylated metabolites. CBD inhibits barbiturate metabolism, increasing barbiturate-induced sleep duration in mice, and phenazone hepatic metabolism [67] due to the inhibition of CYP3A and CYP2C microsomal enzymes [68]. It has been suggested that CBD also induces hepatic CYP3A, CYP2B, and CYP2C [69]. Considering the complexity of CBD pharmacology, available clinical trials address the occurrence of AEs and DDIs in a cohort of healthy subjects [70-77]. Liver safety has been investigated in an open-label DDI trial enrolling 16 healthy subjects aged 18-60 years [76]. Therapeutic oral CBD at a dose of 1500 mg was administered daily for about 3.5 weeks, and the effects on CYP1A2 activity were monitored. Of 16, seven subjects (44%) experienced peak serum alanine aminotransferase values greater than the upper limit of normal. Five subjects (31%) reached the international consensus criteria for drug-induced liver injury. There was no correlation between transaminase elevations and baseline characteristics, CYP2C19 genotype, or CBD plasma concentrations. All alanine aminotransferase elevations above the upper limit of normal began within 2-4 weeks of initial exposure to CBD. DDIs and liver impairment may occur mostly when CBD is co-administered with AEDs, such as clobazam, valproate, and stiripentol, as they share similar metabolic pathways. An open-label DDI study investigated the reciprocal impact on the steady-state pharmacokinetics of CBD, clobazam (and *N*-desmethylclobazam), stiripentol, and valproate; and CBD safety and tolerability when co-administered with each AED. Concomitant cannabidiol had little effect on clobazam exposure, *N*-desmethyl clobazam exposure increased, and stiripentol exposure increased slightly, while no clinically relevant effect on valproate exposure was observed. Concomitant clobazam with CBD increased 7-OH-CBD exposure, without notable 7-COOH-CBD or CBD increases. Stiripentol decreased 7-OH-CBD exposure by 29% and 7-COOH-CBD exposure by 13%. There was no effect of valproate on CBD or its metabolites. CBD was moderately well tolerated, with similar incidences of AEs with concomitant clobazam, stiripentol, or valproate. There were no deaths, serious AEs, pregnancies, or other clinically significant safety findings [77].

A pharmacokinetic DDI trial investigated the effects of repeated dosing of oral CBD on caffeine clearance *via* modulation of CYP1A2 activity in healthy adults. Sixteen healthy subjects received a single 200 mg caffeine dose and placebo on day 1. CBD was then titrated to reach the dosage of 750 mg twice daily through 28 days of treatment. CBD affected the exposure of caffeine, a CYP1A2 substrate, leading to an elevation of 15% for C_max_, 88% for AUC_0-t_, and 95% for AUC_0-∞_, and showed a good safety profile with no unexpected AEs. Diarrhea was the most common, and six subjects discontinued because of elevated liver transaminases [75].

Two studies with a crossover or RCT double-blind design focused on the pharmacokinetic effects of different oral [70] or sublingual formulations of CBD [72]. Fourteen healthy subjects, aged ≥ 18 years (mean 26 [8]), were randomized to take each visit (5 in total) a different CBD formulation: (725 Water soluble. Contains sorbitol; 088 Not water soluble. Contains medium chain triglyceride coconut oil; 126 Water soluble. Contains gum Arabic and maltodextrin; 213 water-soluble. Contains gum Arabic and sorbitol; 625 Not water soluble. Pure CBD as a crystalline powder (>99% purity)) [70]. Comparing the pharmacokinetics of (CBD) and CBD metabolites (6-OH-CBD, 7-OH-CBD, and CBD-COOH) following ingestion of five different CBD formulations, considerable variability was found among the different formulations. When delivered in an aqueous beverage, water-soluble CBD formulations typically evoked the greatest exposure. CBD was not influencing the thermic effect of food; however, it did lower insulin and triglyceride concentrations during the first 30-min following food ingestion. Moreover, a single 30 mg dose of CBD did not influence most of the circulating markers of liver and kidney function. Consistently, no serious AEs were reported [70]. The pharmacokinetic properties and safety profile of a novel CBD wafer formulation compared with an oil formulation were evaluated by Hosseini *et al.* [72]. First, they conducted an open-label, single-dose, crossover study in which 12 healthy volunteers were randomized to receive a sequence of four different single doses of CBD as a sublingual wafer (25 or 50 mg CBD), oil solution (50 mg CBD), or nabiximols oromucosal spray (20 mg CBD, 21.6 mg THC). Then, they carried out a double-blind study where a sublingual wafer (50 mg CBD) was administered twice a day for five days. The extract was generally well tolerated by participants when administered in either wafer or oil form, with some adverse events, including mild or moderate somnolence, sedation, and altered mood. The median maximum concentrations of CBD after administration of the oil solution and wafer were 9.4 and 11.9 ng/mL, respectively. Maximum concentrations of CBD occurred 4 hours after administration, with an estimated terminal elimination half-life of 6 hours [72].

Pharmacokinetic, safety, and tolerability of oral CBD have been evaluated in healthy volunteers after the consumption of different meal compositions. A new lipid oral CBD formulation was administered at doses of 5, 10, and 20 mg/kg in 24 healthy volunteers randomized for each CBD dose, and placebo [73]. This RCT double-blind, placebo-controlled study demonstrated the new CBD formulation is well tolerated even at an escalating dose with no serious AEs reported. AEs of mild or moderate severity were reported by 42% of subjects; the most common being headache (17%) (CBD group 3/18 *versus* placebo group 1/6) and diarrhea (8%) (CBD group 3/18 *versus* placebo group 0/6). The CBD plasma exposure increased in a dose-proportional manner and declined to levels approaching the lower level of quantification by day 8. The terminal elimination half-life was approximately 70 h, suggesting that 2-3 weeks are needed to fully eliminate CBD [73]. Similar findings were found in an open-label crossover study using the highly purified formulation of CBD (Epidiolex). A single CBD dose of 750 mg was administered in thirty healthy subjects following a high-fat/calorie meal (n = 15), a low-fat/calorie meal (n = 14), whole milk (n = 15), or alcohol (n = 14), compared to the fasted state (n = 29). CBD plasma exposure increased under all test treatment conditions in this trial when compared to the fasted state. No serious AEs were recorded. Mild or moderate AEs were reported in 90% (27/30) of subjects. Only two (13%) subjects experienced moderate AEs after administration with alcohol. The most common AEs were headache in 13 (43%) subjects overall, and somnolence and diarrhea in five (17%) subjects overall. Many more subjects were affected by headaches while taking CBD with alcohol (eight (53%) subjects) *versus* the other treatment groups (7%-21% subjects affected). An increased incidence of dizziness was found in subjects while taking CBD with alcohol [71].

Finally, an RCT, double-blind, placebo-controlled trial assessed whether withdrawal symptoms occurred after the abrupt cessation of CBD after prolonged administration in 30 healthy volunteers. No evidence of withdrawal syndrome was found with abrupt discontinuation of short-term treatment with 750 mg CBD twice daily (b.i.d.) for 4 weeks (Part 1) followed by 2 weeks of 750 mg b.i.d. CBD (Part 2, Arm1) or matched placebo (Part 2, Arm2). No serious AEs were reported. Mild or moderate AEs reported were consistent with those reported by other clinical trials [74].

Interestingly, an open-label trial was conducted to assess the pharmacokinetics and safety of CBD in subjects with mild to severe hepatic impairment. The population group consisted of 30 subjects, aged 18-75 years. Alongside CBD, they consumed common concomitant medications, such as beta-blocking agents, diuretics, and drugs for acid-related disorders. The trial consisted of subjects being administered oral CBD 200 mg daily for 3 days. This resulted in common AEs, varying between groups with mild and moderate hepatic impairment. The subject group with mild hepatic impairment had one case of diarrhea and dizziness, while the subject group with moderate hepatic impairment had one case of diarrhea and a low platelet count. However, there was no case of AEs in the subject group of severe hepatic impairment. Neither was an increase in AEs severity or frequency noted with the elevating degree of hepatic impairment [78].

Patients who receive CBD may have co-existing renal disturbances. Therefore, it is important to understand whether dose adjustments are necessary to mitigate the risk of exposure-related toxicity. To gain a deeper insight into the pharmacokinetics, safety, and tolerability of CBD in subjects with mild to severe renal impairment, a phase I, open-label, parallel-group, a single-dose trial was conducted [79]. The subject group was selected to be 32 subjects, ages 18-75 with either mild (n = 8), moderate (n = 8), or severe (n = 8) renal impairment as well as some with normal renal function (n = 8). All groups equally of 8 subjects received 200 mg of oral CBD as a single dose and were followed up after 15 days. In addition to the CBD dose, they consumed concomitant medications including blood pressure-regulating agents (β-blockers, xanthine oxidase inhibitors, calcium channel blockers), thyroid hormones, and diuretics. None were considered to impact the safety or interpretation of the trial data. In the trial outcome, the subjects had defined all AEs as mild. Only five mild AEs were reported by two subjects, one in the mild renal impairment group and one in the normal renal function group. There was no increase in AE frequency or severity with an increasing degree of renal impairment.

### 3.4 Pain Management

CBD has been also evaluated for its analgesic and anti-inflammatory properties. Alaia *et al.* demonstrated that buccally absorbed CBD has good efficacy for pain control right after arthroscopic rotator cuff repair in an RCT, double-blind, placebo-controlled trial. Ninety-nine opioid-naive subjects, aged 18-75, undergoing arthroscopic rotator cuff repair were randomized in placebo (n = 47) and CBD groups (n = 54). For 14 days, subjects received 25 or 50 mg/kg/day CBD alongside opioids as their concomitant medication. One AE of increased transaminase level was experienced in both groups, 4/45 in the CBD group, and 4/47 in the placebo group [80]. Furthermore, a within-subject, randomized, placebo-controlled, double-blind study was carried out to present the analgesic effects, abuse liability, safety, and tolerability of acute CBD [22]. A group of 17 healthy non-cannabis-using subjects, aged 21-50 years were receiving a single dose of 200, 400, or 800 mg oral CBD in a randomized order. With no other concomitant medications reported, all AEs were mild to moderate. There were no differences in AEs between the placebo and CBD groups. Common AEs reported in both groups were lethargy and upset stomach. Subtle mood changes, frequent urination, and wooziness were also reported.

Additionally, an RCT, double-blinded, placebo-controlled trial was performed to examine CBD as an add-on analgesic treatment option for subjects with hand osteoarthritis and psoriatic arthritis [81]. A group of 129 subjects was given synthetic CBD 20 to 30 mg or a placebo daily for 12 weeks. The CBD group comprised 70 subjects with a mean age of 62 years, while the placebo group entailed 66 subjects with a mean age of 61.50 years. The subject group received 10-20 mg/kg/day of either a CBD or placebo tablet orally. Alongside, they also consumed concomitant medication including paracetamol, non-steroidal anti-inflammatory drugs, antileptics, codeine, tramadol, and opioids. Four serious cases of AEs were reported including acute shoulder fracture, malignant hypertension, ductal carcinoma, and lipothymia that were not deemed to be CBD-related. Finally, the therapeutic potential of a topical CBD formulation with shea butter (6.2 mg/mL) has been evaluated for the treatment of pain associated with thumb basal joint arthritis [82]. Eighteen subjects were randomized in a crossover, double-blind, placebo-controlled study design and received topical CBD (6.2 mg/mL CBD with shea butter) or shea butter alone for two weeks. No adverse events were reported, and topical CBD resulted effective in pain control [82].

Since CBD may exert anti-inflammatory and antiviral properties, CBD has been investigated as a potential treatment option for coronavirus disease 2019 (COVID-19). An RCT, double-blind, placebo-controlled trial was carried out for two weeks. The trial involved 109 subjects with mild to moderate symptoms of COVID-19 related [62], subdivided into 49 subjects in the CBD group, who were administered oral CBD at a dosage of 300 mg daily, and 42 subjects in the placebo group. Subjects were taking concomitant medication such as paracetamol, acetaminophen, and dipyrone. Most AEs were mild to moderate. Somnolence, fatigue, decreased appetite, lethargy, weight loss, and diarrhea were commonly reported. These AEs were transient with no statistically significant differences between the placebo and CBD groups.

## 4 DISCUSSION

The therapeutical potential of drugs modulating the activity of endocannabinoid receptors has drawn substantial interest [23]. Since the approval of the highly purified formulation of CBD, Epidiolex, in 2018, a growing number of studies were conducted to investigate the efficacy and the safety of CBD in several medical conditions. We previously reviewed the literature regarding CBD pharmacokinetic properties, tolerability, and safety profile in both preclinical and clinical studies [28]. The main aim of the present work was to offer an updated review of the available evidence regarding the use of CBD in the clinical context.

Compared to our previously published article [28], new data have become available on the long-term efficacy and tolerability of CBD for the treatment of TREs suggesting that CBD has a good safety profile in both children and adults’ populations. Interestingly, in the last few years, a larger number of clinical trials have been conducted for multiple clinical indications, outside of LGS and DS, including psychosis, anxiety disorders, addictions, and pain management. Moreover, many ongoing clinical trials are evaluating the efficacy and the safety profiles of CBD in conditions such as Alzheimer’s disease, several forms of chronic pain, fibromyalgia, developmental and behavioral disorders, and liver and heart disorders in an effort to better understand CBD pharmacodynamics and pharmacokinetics, and its therapeutic potential. Overall, this updated systematic review provides new evidence confirming a well-tolerated safety profile and suggesting that CBD may represent a new therapeutic approach as a stand-alone or an add-on drug for several unmet clinical needs.

Across all included trials, most patients reported the occurrence of at least one AEs. Most AEs were mild or moderate in severity. The few serious AEs reported were frequent causes of treatment discontinuation or hospitalization with a full recovery after CBD interruption. CBD potently inhibits hepatic metabolism by acting on CYP3A4 and CYP2C19. These enzymes also metabolize several AEDs that are often taken concomitantly with CBD, such as clobazam, valproate, and stiripentol, in a cohort of patients enrolled in clinical trials focusing on TRE. Oral CBD, when prescribed at higher doses (10-20 mg/kg/day), was associated with an increased rate of serious AEs, among which elevation of liver transaminase, somnolence, sedation, and respiratory infections were most commonly reported. The majority of cases with increased liver transaminase were in children or adults with epilepsy who were also being treated with sodium valproate. The exact mechanism of this interaction is not fully understood. CBD and valproate co-administration does not significantly alter their plasma levels or of their metabolites [77]. However, CBD metabolite 7-COOH-CBD and valproate and its metabolite 4-ene-valproic acid may affect hepatic mitochondrial function. Similarly, concomitant CBD did not significantly affect clobazam plasma exposure in healthy subjects [77]. Given CBD complex and multimodal pharmacology with a potential increase of side effects when taken with clobazam, two metanalyses evaluated the efficacy and tolerability of CBD with or without concomitant clobazam, providing conflicting results. Indeed, a metanalysis of four phase III, RCTs, double-blind, placebo-controlled trials (GWPCARE1, 2, 3, 4) [83] found that the safety profile of oral highly purified CBD (10 or 20 mg/kg/day for a 14-week treatment) was affected by the concomitant administration of clobazam. Reported AEs and serious AEs were more frequent in patients taking both CBD and clobazam than in those taking CBD without clobazam and placebo. Somnolence was frequently reported by patients with concomitant clobazam and increased at higher CBD doses [83]. Conversely, Gunning *et al.* did not find an increased incidence of AEs in LGS and DS patients taking concomitant clobazam [84]. Somnolence was the most frequent AE reported in 22% of patients with LGS and 27% of patients with DS, of whom 67% and 84%, respectively, were taking concomitant clobazam, and was not considered dose related. Other common AEs, such as diarrhea, decreased appetite, and vomiting were not more common in patients on clobazam. Serious AEs related to CBD and their possible relation to DDIs were analyzed by a systematic review of RCTs involving the administration of oral CBD for at least one week in healthy subjects and in clinical populations, including patients with epilepsy, PD, social anxiety disorder, and psychosis [85]. A higher incidence of serious AEs was observed in epilepsy RCTs. Somnolence, sedation, the elevation of liver transaminase level, skin rash, and respiratory disturbances were the most commonly reported serious AEs. These effects seem to be related to DDIs, especially with valproate in the case of increased liver transaminase level and with clobazam in the case of sedation and somnolence. No significant serious AEs were found in studies conducted on healthy subjects or patients with PD, Huntington’s disease, type 2 diabetes, Crohn’s disorder, and social anxiety disorders, even considering the concomitant medications [85]. These findings were further confirmed by a systematic review and metanalysis of CBD AEs across all medical indications suggesting that CBD is well tolerated and induces few serious AEs, whose rate increases when combined with other drugs in childhood epilepsy populations [86]. Finally, the pharmacokinetic interaction between CBD and mTOR inhibitors has been evaluated only in one retrospective chart review. The plasma level of everolimus and sirolimus increased significantly when taken with CBD [44]. However, the overall severity and prevalence of AEs were similar to those identified in RCTs. Further research and clinical studies should evaluate the pharmacokinetic interactions of CBD with several classes of medications, as the clinical indication of CBD is expanding. New therapeutic drug monitoring methods should be developed to help clinicians in the clinical management of patients. Indeed, more accurate detection of CBD and its metabolites may help the dose adjustments and reduce the rate of AEs especially when CBD is co-administered with other drugs [87, 88].

Critical factors to evaluate CBD safety and tolerability are the dose, the route of administration, and the frequency of use. In the included trials, the CBD dose ranged from 2.5 to 50 mg/kg/day as a single or multiple administration. Importantly, compared to our previous review on CBD toxicity and AEs [28], more data are available on long-term CBD use, particularly in clinical trials in epileptic children and adults for up to 36 months. Moreover, clinical studies have focused on CBD antiseizure properties in other forms of refractory childhood epilepsy, expanding its therapeutic application.

Only three-open label studies aimed at evaluating CBD effects on cognition. Oral CBD, at the common dosages used in clinical trials, seems to not affect cognitive functions. These preliminary findings may pave the way for an expansion of CBD clinical indications. Interestingly, CBD has recently been under investigation as a potential add-on treatment for neurological disorders, such as PD and AD. In a study involving PD patients with rapid eye movement behavior disorder, although CBD was ineffective to reach the primary outcome, it showed a good safety profile with no significant AEs compared to a placebo. Similar findings were reported by studies investigating the therapeutic role of CBD in the adult cohort population with anxiety disorders, addictions, and chronic pain conditions.

Most of the included studies have used the highly purified formulation of CBD Epidiolex that is administered orally. Few well-designed clinical trials are still available on different formulations and routes of administration. In studies using a transdermal gel formulation, CBD dose ranged from 50 to 500 mg/day. Most patients experienced at least one AE of mild or moderate severity. No significant serious AEs were reported. The most common AEs were gastrointestinal and respiratory disturbances and somnolence. An increased rate of skin rash compared to studies using oral CBD was reported. Only single, open-label studies evaluated different formulations of CBD, including smoking CBD cigarettes, liquid spray, and sublingual wafers, reporting an acceptable safety profile. However, further well-designed and controlled trials are needed to support the therapeutic use of these CBD formulations for medical purposes.

A concerning issue in the CBD field is the availability of over-the-counter (OTC) CBD products, which have not been evaluated in clinical trials. OTC preparations may contain CBD at different doses along with other cannabinoids and/or other contaminants [89-91]. This issue requires more research, as the market of OTC CBD products is poorly regulated.

## CONCLUSION

CBD has shown a well-tolerated safety profile with few AEs. However, its ability to inhibit the hepatic metabolism of other drugs, such as clobazam, valproate, and stiripentol, can potentially cause AEs. Indeed, an increased rate of AEs, serious AEs, and discontinuation of treatment has been demonstrated in several clinical trials focusing on TRE in children. Data from clinical studies involving adult subjects with disease conditions are still limited. Therefore, additional data on CBD tolerability and safety from clinical studies outside childhood epilepsy syndromes, studies involving subjects with different medical conditions, and studies of over-the-counter CBD products are needed.

## Figures and Tables

**Table 1:** Cannabidiol adverse events in clinical studies.

**Study Design**	**Population**	**CBD Dose; Administration Route**	**Time**	**Concomitant Medication**	**Reported AEs**	**Authors**
**Pharmacokinetics and Drug-Drug Interaction**						
RCT, crossover, placebo-controlled trial	Age ≥ 18 (mean 26 [8]); 14 healthy subjects randomized to take at each visit a different CBD formulation: (725 Water soluble. 088 Not water soluble. 126 Water soluble. Contains gum Arabic and maltodextrin 213 water-soluble. Contains gum Arabic and sorbitol 625 Not water soluble. Pure CBD as a crystalline powder (>99% purity)	30 mg; oral	Single dose	Not reported	No AEs related to liver or kidney dysfunction	Abbotts *et al.,* 2022 [70]
Open-label, crossover trial	Age 18-60; 30 healthy subjects: high-fat/calorie meal (n = 15), a low-fat/calorie meal (n = 14), whole milk (n = 15), or alcohol (n = 14), relative to the fasted state (n = 29).	750 mg; oral	Single dose; days follow-up	None	AEs mild/moderate in 27/30 (90%); moderate AEs after administration with alcohol (n = 2 (13%)): headache (n = 13 (43%)), somnolence and diarrhea (n = 5 (17%)); headache more frequent when taking CBD with alcohol (n = 8 (53%)) *versus* other groups (7%-21%); dizziness more frequent when taking CBD with alcohol (n = 3 (20%))	Crockett *et al.,* 2020 [71]
Open-label, four-way crossover trial (*Single dose study*) RCT, double-blind, placebo-controlled trial (*Multiple-dose study*)	*Single-dose study*: Age 18-50 (mean 33 [32-49]); 12 healthy subjects randomized to receive a sequence of four different CBD single doses as a sublingual wafer (25 or 50 mg CBD), oil solution (50 mg CBD), or nabiximols oromucosal spray (20 mg CBD, 21.6 mg THC) *Multiple-dose study:* Age 18-50 (mean 30 [27-34]); 6 healthy subjects randomized in ratio 4:2 to receive CBD or placebo.	*Single-dose study:* 25 or 50 mg CBD; sublingual 50 mg in oil; oral 20 mg CBD/21.6 mg THC in liquid spray; oromucosal *Multiple-dose study:* 50 mg CBD twice daily; sublingual4	*Single-dose study:* 1 day *Multiple-dose study:* 5 days	Not reported	AEs mild/moderate *Single-dose study:* somnolence (k = 5 in n = 4 (17%)), sedation (k = 2 in n = 2 (8%)), altered mood (k = 2 in n = 2 (8%)) in sublingual and oral CBD groups *Multiple-dose study:* 1 participant withdrew from treatment due to altered mood but remained in the study	Hosseini *et al.,* 2021 [72]
Open-label, fixed-single sequence trial	Age 18-55 (mean range from 26.2 to 35.1); 78 healthy subjects: 12 clobazam + CBD; 12 CBD + clobazam; 12 stiripentol + CBD; 12 CBD + stiripentol; 12 valproate + CBD; 14 CBD + valproate	750 mg twice daily; oral	3-25 days	Clobazam, stiripentol, valproate	AEs mild/moderate: 7/77 withdrawal: 1 withdrew consent to participate; 6 (7.8%) discontinuations due to AEs. Most common AE was rashes (5 of 6) in subjects taking clobazam concomitantly with steady-state cannabidiol (2 subjects) or valproate concomitantly with steady-state cannabidiol (3 subjects)	Morrison *et al.,* 2019 [77]
RCT, double-blind, placebo-controlled trial	Age 18-48; 24 healthy subjects assuming CBD after a high-fat meal: Cohort 1 (5mg/kg CBD), n = 6 CBD or n = 2 placebo; cohort 2 (10mg/kg CBD), n = 6 CBD or n = 2 placebo (cohort 2); cohort 3 (20mg/kg CBD), n = 6 CBD or n = 2 placebo (cohort 2)	5, 10, 20 mg/kg/day; oral	Single dose, 15 days follow-up	None	AEs mild/moderate in 42% (10/24). Most common: headache (17%) (CBD group 3/18 *versus* placebo group 1/6) and diarrhea (8%) (CBD group 3/18 *versus* placebo group 0/6)	Perkins *et al.,* 2020 [73]
RCT, double-blind, placebo-controlled trial	Age 18-45 (mean 25.3 [6.7]). 30 healthy subjects were randomized to receive 750 mg CDB twice daily (b.i.d.) for 4 weeks (Part 1) followed by 2 weeks of 750 mg b.i.d. CBD (Part 2, Arm1) or matched placebo (Part 2, Arm2) to assess the occurrence of withdrawal symptoms induced by abrupt cessation of CBD after prolonged administration	750 mg twice daily; oral	4-6 weeks	Not reported	Part 1 (CBD only): AEs in 97% (29/30); most common AEs diarrhea (63%), headache (50%), abdominal pain (47%), nausea (43%), and fatigue (33%); 4 (13%) severe AEs, and 6 (20%) moderate AEs. Discontinuation in 30% (9/39) in Part 1 because of AEs due to skin rash (7 (23.3%)), liver disorder (2 (6.7%)), and eosinophilia (1 (3.3%)). Part 2, Arm 1 (CBD): AEs in 67% (6/9) Part 2, Arm 2 (placebo) AEs in 75% (9/12); Increased incidence of headaches in Arm2 (7 (58%) in Arm2 *vs*. 2 (22%) in Arm 1). All AEs mild in intensity were comparable between Arm 1 and Arm 2. Most common AEs in Arm 1: diarrhea (4 (44%)), nausea, and headache (2 ((22%)); in Arm2, headache (7 (58%))	Taylor *et al.,* 2020 [74]
Open-label, fixed single-sequence trial	Age 18-60 (mean 32.6 (12.9)); 16 healthy subjects	250 mg/day-750 mg twice daily; oral	28 days	Caffeine	AEs moderate/mild occurred in 87.5%. (14/16). Most common AEs: is diarrhea. Elevated liver transaminase levels occurred in one subject. Six subjects (37.5%) were discontinued from the trial drug because of AEs: 3 increased ALT and (AST) ; 1 increased ALT and AST and eosinophilia; 1 increased ALT, abdominal discomfort, vomiting, and increased percentage of eosinophils; and 1 increased ALT and AST, nausea, and syncope	Thai *et al.,* 2021 [75]
Open-label, fixed single-sequence trial	Age 18-60 (mean 29 [range from 23.5 to 37.8]); 16 healthy subjects	1500 mg/day; oral	27 days	Acetaminophen, oral contraceptive	AEs mild/moderate in 88% (14/16). Most common diarrhea in eight (50%) participants and abdominal discomfort in five (31%) participants. Peak serum alanine aminotransferase (ALT) values greater than the upper limit of normal (ULN) in 44% (7/16); for five (31%) participants, the value exceeded 5 × ULN	Watkins *et al.,* 2021 [76]
Open-label trial	Age 18-75 (mean range from 52.7 to 57.5); 30 subjects: 1 of 4 subject groups (mild [n = 8], moderate [n = 8], or severe [n = 6] hepatic impairment or normal hepatic function [n = 8])	200 mg; oral	3 days	Most common: beta-blockers agents, diuretics, drugs for acid-related disorders	AEs moderate/mild. In the mild hepatic impairment group AEs occurred in 12.5% (1/8): 1 diarrhea, dizziness; in the moderate hepatic impairment group AEs occurred in 12.5% (1/8): 1 diarrhea, 1 low platelet count; no AEs in the severe hepatic impairment group. There was no increase in AE frequency or severity with increasing degrees of hepatic impairment	Taylor *et al.,* 2019 [78]
An open-label, parallel-group trial	Age 18-75 (mean from 58.8 to 64.6 years); 32 subjects with mild [n = 8], moderate [n = 8], or severe [n = 8] renal impairment or normal renal function [n = 8]	200 mg; oral	Single dose, 15 days follow-up	Beta-Blockers, xanthine oxidase inhibitors, calcium channel blockers, thyroid hormones, diuretics	All AEs mild. Only five mild AEs were reported by two subjects 1 in the mild renal impairment group and 1 in the normal renal function group. There was no increase in AE frequency or severity with increasing degree of renal impairment	Tayo *et al.,* 2020 [79]
**Neurology**						
RCT, double-blind, placebo-controlled trial	Age 16-55 (mean 29.5, range from 17.4 to 54.5); 35 subjects with epilepsy already receiving a stable dose of stiripentol or valproate randomized in a 4:1 ratio to receive concomitant CBD or placebo. **Stiripentol group:** 14 patients (2 placebo; 12 CBD); **Valproic acid group**: 20 patients (4 placebo; 16 CBD; one withdrawal before treatment).	20 mg/kg/d; oral	10-days, followed by a 14-day maintenance period	Stiripentol, valproic acid, lacosamide, clobazam, ethosuximide, lamotrigine, topiramate, levetiracetam, rufinamide, zonisamide, clonazepam, lorazepam, oxcarbazepine, caramazepine	**Stiripentol group:** AEs mild in 8/12 (67%) all in CBD patients: diarrhea and fatigue, severe rash leading to withdrawal (n = 1 (8%)), increased transaminase (n = 2 (17%)) **Valproic acid group:** AEs mild/moderate in 14/16 (88%) in the CBD group and 1/4 (25%) in the placebo group: diarrhea, 1 increased transaminase level	Ben-Menachem *et al.,* 2020 [50]
Cohort study	Age 2-16 (mean 10.5); 50 subjects with drug-resistant EE	2-5 mg/kg/day; oral	1 year	Not reported	AEs mild/moderate: drowsiness (k = 16 (32%)), decreased appetite (k = 15 (30%)), diarrhea (k = 14 (28%)), irritability or behavior disturbances (k = 12 (26%)), weight loss (k = 10 (20%)), vomiting (k = 7 (14%)), mood changes (k = 3 (6%)), insomnia (k = 5 (10%)), blurred vision (k = 1 (2.6%)), dry mouth (k = 1 (2.6%)), fever (k = 1 (2.6))	Caraballo *et al.,* 2020 [39]
RCT, double-blind, placebo-controlled trial	Age ≥ 18 (mean 57); 36 subjects with Parkinson's disease and comorbid REM behavior disorder (RBD): 20 CBD group, 16 placebo group	75-300 mg daily in a fixed progressive dose scheme: 75 mg, 1^st^ week; 150 mg, 2^nd^ week; 300 mg, 3^rd^ week until the 12^th^ week of treatment; oral	12 weeks	Antidepressants, clonazepam, melatonin	No differences between CBD and placebo groups: epigastric pain (n = 1/20 (5%)), headache (n = 1/20 (5%)), drowsiness (n = 1/20 (5%)), sadness (n = 2/20 (10%)), dizziness (n = 1/20 (5%)) in CBD group	de Almeida *et al.,* 2021 [24]
OLE trial GWPCARE5 (NCT02224573)	Age 2-55 (mean 9.8 [4.4]); 264 subjects with DS	2.5-30 mg/kg/day, mean modal dose 21 mg/kg/day; oral	274 days	Clobazam, valproic acid, stiripentol, levetiracetam, topiramate	AEs mild/moderate in 246/264 (93%) and serious in 77/264 (29%): diarrhea (n = 91 (34%)), decreased appetite (n = 67 (25.4%)), somnolence (n = 65 (25%)), nasopharyngitis (n = 41 (16%)), vomiting (n = 37 (14%)), upper respiratory tract infection (n = 36 (14%)), fatigue (n = 27 (10%)), status epilepticus (n = 29 (11%)), convulsion (n = 40 (15%)), pyrexia (n = 72 (27%)), pneumonia (n = 7 (3%)), aspartate aminotransferase increased (n = 5 (2%)), dehydration (n = 4 (2%)), influenza (n = 4 (2%)), generalized tonic-clonic seizure (n = 4 (2%))	Devinsky *et al.,* 2019 [47]
Retrospective chart review	Age 3-45 (mean 17); 25 subjects with TSC	5-20 mg/kg/day; oral	Not reported	Sirolimus, everolimus, bupropione, felbamate, lamotrigine, quetiapine, trazodone, lysine, taurine, topiramate, zonisamide, clobazam, rufinamide, bivaracetam, risperidone, lacosamide, vigabatrin, clonazepam	AEs in 10/25 (40%): diarrhea (n = 3/25 (12%)); drowsiness, increased and severe mouth sores, increased acne, ankle swelling, sinusitis, abdominal pain, mild elevation of transaminases and increased phenytoin level	Ebrahimi-Fakhari *et al.,* 2020 [44]
Open-label trial	Mean age 20.8 (15) years (10 [5] years for children and 33 [14] years for adults); 169 subjects with TRE: 89 children, 80 adults	5-50 mg/kg/day; oral	2 years	Clobazam, valproate	AEs mild/moderate: 3042 AEs (severe in 44/3042), of which 1998 (971 children, 1027 adults) CBD-related: diarrhea (k = 737/3042), sedation (k = 391/3042), decreased appetite (k = 107/3042), increased liver function tests (k = 21/3042 in n = 11 (6.5%), of which n = 5 (45%) were also taking valproate), death (n = 1 (1%), not CBD-related); 109 serious AEs (48 children, 61 adults), of which 27 CBD-related: hospital admissions, skin rashes	Gaston *et al.,* 2021 [32]
Open-label trial	Age 2-16 (mean 8); 9 subjects with ES	25, 45, 50 mg/kg/day; oral	12 months	Low glycemic treatment, valproate, vigabatrin	AEs mild in 8/9 (89%): drowsiness (n = 7 (78%)), diarrhea (n = 2 (22%)), ataxia (n = 2 (22%)), appetite loss (n = 2 (22%)), elevated liver enzymes (n = 1 (11%)), agitation (n = 2 (22%)), twitchiness (n = 1 (11%)), irritability (n = 1 (11%))	Herlopian *et al.,* 2020 [34]
Retrospective chart review	Age 2-18 (mean 5.1, range from 1.2 to 15.8); 34 subjects with LGS and 10 subjects with DS	10-20 mg/kg/day; oral	6 months	Antiepileptics: most frequent valproate, levetiracetam	Gastrointestinal problems (mild liver enzyme elevation, vomiting, and diarrhea; n = 7 (16%)), behavioral changes (irritability, hyperactivity, excessive alertness, sleep disturbances; n = 5 (11.4%)), increased seizure frequency (n = 2 (5%)) in LGS and DS groups; AEs leading to discontinuation: drowsiness (n = 1 (2%)), gastrointestinal problems with acute pancreatitis (n = 1 (2%)), and behavioral changes (n = 1 (2%))	Koo *et al.,* Oct 2020 [45]
Open-label, multicenter trial	Age (mean 12.8 [1.7-51]); 152 subjects: 94 with LGS, 54 with DS; Age (mean 13.3[0.4-62.1]); 455 subjects with other TRE	2-50 mg/kg/day; oral	144 weeks	Clobazam, felbamate, lamotrigine, levetiracetam, rufinamide, stiripentol, topiramate, valproic acid	AEs mild/moderate. AEs in 91% LGS/DS group, of whom 41% reported serious AEs. Most common: somnolence (30%), convulsion (24%), and diarrhea (24%). Most common all-cause serious AEs: convulsion (14%), status epilepticus (9%), pneumonia (5%), and pyrexia (4%). Increased liver transaminase in 15% (22/152), of these 82% (18/22) reported concomitant valproate. Somnolence in 38% (38/101) subjects taking concomitant clobazam, and in 18% (9/51) subjects not taking clobazam. 12 deaths reported were deemed not-treatment-related by the investigator	Laux *et al.,* 2019 [31]
Open-label trial	Age ≥ 19 (mean 34 [14]); 27 subjects with TRE	Mean modal dose of 36.5 mg/kg/day; oral	1 year	Not reported	Not reported	Martin *et al.,* 2019 [33]
Open-label trial	Age 0-59 (mean 16.7 [13.4]); 48 subjects with TRE	4-25 mg/kg/day; oral	3 months	Antiepileptics	No significant AEs related to add-on CBD treatment	Metternich *et al.,* 2021 [30]
RCT, double-blind, placebo-controlled trial (GWPCARE2)	Age 2-18; 199 patients with DS: n = 67 10mg/kg/d CBD10 group; n = 67 20 mg/kg/d CBD20 group; n=65 placebo	10-20 mg/kg/day; oral	14 weeks	Antiepileptics: most common valproate, clobazam	AEs moderate/mild occurred in 176 of 198 patients (88.9%): 58 of 65 (89.2%) in the placebo group, 56 of 64 (87.5%) in the CBD10 group, and 62 of 69 (89.9%) in the CBD20 group. Common AEs decreased appetite, diarrhea, somnolence, pyrexia, and fatigue. Elevated liver transaminase levels occurred more frequently in the CBD20 (n = 13) than the CBD10 (n = 3) group, with all affected patients given concomitant valproate sodium	Miller *et al.,* 2020 [51]
RCT, double-blind, placebo-controlled, multicenter trial (STAR 1), with OLE (STAR 2)	Age 18-70 (mean 39.2 [12.78]); 188 subjects with drug-resistant focal epilepsy receiving a stable regimen of up to 3 antiseizure medications randomized in a ration 1:1:1 (195mg CBD, 63 participants; 390mg CBD, 62 participants; placebo, 63 participants)	195, 390 mg; transdermal	12 weeks, followed by an open-label extension study for up to 2 years	Three concomitant antiepileptics	AEs in 50.4% (63/125) CBD group, and in 41.3% (26/63) in the placebo group. Similar rates in the CBD groups. Discontinuation in 7% (14/188) due to Aes	O'Brien *et al.,* 2022 [48]
Open-label, multicenter trial	Age 1-18 (mean 10.4); 50 subjects with TRE	25-50 mg/kg/day, mean modal dose of 35 mg/kg/day; oral	36 months	Clobazam, levetiracetam, lamotrigine, topiramate zonisamide, rufinamide oxcarbazepine, phenytoin, lacosamide phenobarbital, vigabatrin, ethosuximide, perampanel, valproic acid, prorenata (as needed)	All AEs mild/moderate; Common AEs: upper respiratory infection, gastrointestinal disorders, pyrexia, somnolence	Park *et al.,* 2020 [42]
OLE trial of the RCT, placebo-controlledtrial GWPCARE5 (NCT02224573)	Age 1-65 years (mean 15.9 [9.5]); 366 subjects with LGS	5-30 mg/kg/day; mean modal dose of 24 mg/kg/day; oral	Mean of 826 days (range, 3-1421)	Clobazam, valproate, lamotrigine, levetiracetam, rufinamide	AEs in 96% of patients, serious AEs in 42%, and AE-related discontinuations in 12%. Common AEs were convulsion (39%), diarrhea (38%), pyrexia (34%), and somnolence (29%). 55 (15%) patients experienced liver transaminase elevations; 40 (73%) were taking concomitant valproate	Patel *et al.,* Jun 2021 [40]
Open-label trial	Age 1-18 (mean range 1.9-16.3 [4.7]); 29 subjects with DEE	2-25 mg/kg/day; oral	Minimum 6 months	Clobazam	All AEs mild/moderate; Common AEs: somnolence, decreased appetite and diarrhea	Pietrafusa *et al.,* 2019 [38]
Open-label trial	Age 3-18 (mean 10.5 [3.8]); 48 subjects, Age: 3-18 years with DEE	125-500 mg; transdermal	6.5 months	Valproate, clobazam, levetiracetam, lamotrigine, topiramate	All AEs mild/moderate in 48 of 48 subjects; Common AEs: upper respiratory tract infection (n = 42 (42%)), nasopharyngitis (n = 21 (10%)), somnolence (n = 13 (6%)), vomiting (n = 10 (5%)), application site dryness (n = 8 (4%)), application site pain (n = 8 (4%)), decreased appetite (n = 8 (4%)), diarrhea (n = 8 (4%)), gastroenteritis (n = 8 (4%)), pyrexia (n = 8 (4%)), viral infection (n = 8 (4%)), viral upper respiratory tract infection (n = 8 (4%)), contusion (n = 6 (3%)), cough (n = 6 (3%)), ear infection (n = 6 (3%)), fall (n = 6 (3%)), fatigue (n = 6 (3%)), pneumonia (n = 6 (3%)), scratch (n = 6 (3%)), seizure (n = 6 (3%))	Scheffer *et al.,* 2021 [37]
OLE trial GWPCARE5 (NCT02224573)	Age 2-18 (mean 9.7 [4.4]); 315 subjects with DS	2.5-30 mg/kg/day; mean modal dose of 22 mg/kg/day; oral	444 days (range = 18-1535)	Three concomitant antiepileptics	AEs occurred in 97% (306/315) of patients (mild, 23%; moderate, 50%; severe, 25%). The most common AEs: diarrhea (43%), pyrexia (39%), decreased appetite (31%), and somnolence (28%). 28 (9%) patients discontinued due to AEs. 69 (22%) liver transaminase elevations, 84% were on concomitant valproic acid	Scheffer *et al.,* 2021 [52]
OLE trial GWPCARE5 (NCT02224573)	Age 2-55 (mean 15.9 [9.5]); 66 subjects with LGS	2.5-30 mg/kg/day; mean modal dose of 23 mg/kg/day; oral	38 weeks	Clobazam, valproic acid	AEs (92.1%): mild/moderate; Common AEs: Diarrhea (26.8%), somnolence (23.5%), convulsion (21.3%), Liver transaminase elevations (10.1%)	Thiele *et al.,* 2019 [41]
OLE trial of the RCT, placebo-controlled trial GWPCARE6 (NCT02544763)	Age 1-65 (mean age 13.5 [10.5]); 199 TSC associated seizures subjects: 75 placebo; 64 CBD 25mg/kg/d; 60 CBD 50mg/kg/d	5-50 mg/kg/d; mean modal dose of 27 mg/kg/day; oral	Mean of 267 days (range, 18-910)	Valproate, vigabatrin, clobazam, levetiracetam, lamotrigine, lacosamide, oxcarbazepine	AEs in 92% of patients (184/199): most AEs were mild or moderate severity. Most common AEs were diarrhea (42%), seizure (22%), and decreased appetite (20%). AEs led to permanent discontinuation in 6% of patients. One death was deemed not-treatment-related by the investigator. Elevated liver transaminases occurred in 17 patients (9%) patients; 12 were taking valproate	Thiele *et al.,* 2021 [46]
RCT, double-blind, parallel-controlled, multicenter trial (GWPCARE6) (NCT02544763)	Age 1-65 (mean 11.4 [1.1-56.8]); 224 subjects with TSC and TRE randomized in 76 placebo; 75 CBD 25mg/kg/d; 73 CBD 50mg/kg/d	25 or 50 mg; oral	16 weeks: 4 weeks titration phase, 12 weeks maintenance phase, up to 10 days taper phase	Valproate, vigabatrin, clobazam, levetiracetam, lamotrigine, everolimus	AEs were reported by 70 patients (93%) in the CBD25 group, 73 patients (100%) in the CBD50 group, and 72 patients (95%)in the placebo group; 88% of AEs were mild or moderate. Most common AEs: diarrhea (placebo group, 19 (25%); CBD25 group, 23 (31%); CBD50 group, 41 (56%)), somnolence (placebo group, 7 (9%); CBD25 group, 10 (13%); CBD50 group, 19 (26%)), occurred more frequently with CBD than placebo. Discontinuation because of AEs 8 in the CBD25 group, 10 in the CBD50 group, and 2 in the placebo group. Elevated liver transaminase levels in 28 subjects (18.9%) taking CBD *vs.* none taking placebo	Thiele *et al.,* 2021 [43]
Open-label trial	Age 3-19 (mean 14); 38 subjects with TRE	5-50 mg/kg/day; oral	1 year	Not reported	No significant AEs related to add-on CBD treatment	Thompson *et al.,* 2020 [36]
RCT, double-blind, placebo-controlled trial	Age 18-65 (mean 36.6 CBD group; 37.6 placebo); 20 patients with uncontrolled epilepsy on stable doses of CLB randomize in a ratio 4:1 to receive CBD or placebo	20 mg/kg/day; oral	32 days: 1-10 day titration phase, 11-32 maintenance phase	Clobazam	CBD group: most AEs were mild (5 (31.3%)) or moderate (7 (43.8%)), and 1 (6.3%) was severe (seizure cluster leading to withdrawal); 2 (12.5%) increased transaminase level with concomitant valproate; 3 (18.9%) reduction of CBD dose due to occurrence of rash, diarrhea, and multiple events of sedation, slurred speech, and word-finding difficulties. Placebo group: all AEs were mild. Most common AEs overall: diarrhea, nausea, vomiting, sedation, somnolence	VanLan-dingham *et al.,* 2020 [49]
Open-label trial	Age 1-17 (mean 7.6); 61 subjects with TRE	5-40 mg/kg; oral	2 months	Clobazam	Most AEs mild/moderate; Common AEs: somnolence (21.3%), anemia (18.0%), diarrhea (16.4%), Less common SAEs: thrombophlebitis, apnea, and skin rash	Wheless *et al.,* 2019 [56]
Open-label trial	Age 12-25; 31 subjects with treatment-resistant anxiety disorders	800 mg; oral	12 weeks	SSRI, SNRI, tricyclic antidepressants, tetracyclic antidepressants, fluoxetine, citalopram, sertraline, mirtazapine, venlafaxine, desvenlafaxine, dosulepin	AEs mild/moderate (80.6%): fatigue, low mood, hot flushes, cold chills	Berger *et al.,* 2022 [63]
Open-label trial	Age mean 33.6; 120 frontline healthcare workers with COVID-19 to reduce burnout and exhaustion symptoms	300 mg; oral	28 days	Not reported	Most AEs mild/moderate (>10%): somnolence (n = 34/120 (29%)), fatigue (n = 27/120 (23%)), increased appetite (n = 19/120 (16%)), diarrhea (n = 13/120 (11%)), weight gain (n = 12/120 (10%)), lethargy (n = 12/120 (10%)); less common AEs elevated liver enzymes (critical n = 1/120 (1%), mild n = 3/120 (2%)), pharmacodermia (n = 1 (1%))	Crippa *et al.,* 2021 [66]
RCT, double-blind, placebo-controlled trial	Age mean 32.9 (6.8); 31 subjects with cocaine use disorder: 14 CBD group; 17 placebo group	300mg; oral	10 days	Not specified	AEs mild/moderate. Sleepiness and increased sleep duration in 5/14 of CBD group and 3/17 of control group; nausea in 2/14 CBD group and 1/17 of control group; headache in 2/14 CBD group and 1/17 of the control group. No serious AEs.	de Meneses-Gaya *et al.,* 2021 [61]
RCT, double-blind, placebo-controlled trial	Age 8-16 (range from 11 to 16.9); 8 children with intellectual disability and severe behavioral problems randomized 1:1 in CBD (4) or placebo (4) group	20 mg/kg/day, maximum dose 500 mg, twice daily; oral	8 weeks with 9 days titration period	Not reported	AEs mild/moderate (all n = 1 (25%) if not specified otherwise): eyes rolled up, tics/grimace, ear ringing, drooling/pooling, abdominal pain, decreased appetite, increased appetite, constipation, decreased weight, increased weight), restlessness/pacing/cannot sit still, jitter/jumpiness/nervousness, acne, urination incontinence/nocturnal enuresis, crying/feelings of sadness, drowsiness/lethargy/sedation, excessive sleep, insomnia in CBD group; headache, nose congestion/running nose, increased appetite, crying/feelings of sadness, insomnia, increased weight (n = 3 (75%)) in the placebo group	Efron *et al.,* 2021 [59]
Retrospective, open-label case series	Age (mean 39.1 [17.39]); 11 subjects with PTSD	22-28 mg in capsules or 425-575 mg in liquid spray; oral	8 weeks	Antiepileptics, antidepressants, antipsychotics, anxiolytic/sedatives, beta-blockers	AEs in 5/21 (24%): gastrointestinal bloating or pain (n = 2 (10%)), fatigue (n = 2 (10%)), daytime fogginess (n = 1 (5%)), impaired concentration (n = 1 (5%)), worsening reflux (n = 1 (5%))	Elms *et al.,* 2019 [65]
RCT, double-blind, placebo-controlled trial	Age 16-60 (range from 18.35 to 43.35); 48 subjects with Cannabis Use Disorder (CUD) were randomized into 12 placebo groups, 12 CBD 200mg group, 12 CBD 400 mg group, and 12 CBD 800mg group	200, 400, 800 mg; oral	4 weeks	Not reported	AEs mild/moderate; no differences between placebo *versus* CBD 200, 400, 800 mg CBD: 65 mild and 9 moderate AEs in the placebo group; 42 mild and 4 moderate AEs in CBD 200 mg group, 96 mild and 8 moderate AEs in CBD 400 mg group, 78 mild and 8 moderate AEs in the CBD 800 mg group	Freeman *et al.,* 2020 [57]
RCT, double-blind, placebo-controlled trial	Age 21-65 (mean 49.8 [9.2]); 50 drug-abstinent subjects with heroin use disorder was assessed to test acute (1 hour, 2 hours, and 24 hours), short-term (3 consecutive days), and protracted (7 days after the last of three consecutive daily administrations) effects of CBD administration (400 or 800 mg, once daily for 3 consecutive days) on drug cue-induced craving and anxiety	400, 800 mg; oral	Acute (1, 2, and 24 h), short-term (3 consecutive days), and protracted (7 days after the last of 3 consecutive daily administrations)	Not reported	No difference between CBD and placebo groups: diarrhea (n = 1 (4%)), headache (n = 1 (4%)), tiredness or fatigue (n = 1 (4%)) in the CBD group; diarrhea (n = 2 (13%)) headache (n = 2 (13%)), tiredness or fatigue (n = 1 (7%)) in the placebo group	Hurd *et al.,* 2019 [60]
Open-label trial	Age 18-65 (mean 35.1 [10.58]); 31 acutely psychotic subjects with comorbid tobacco use disorder (16 CBD-group; 15 placebo)	20 mg/cigarette; smoking	0-28 days	Antipsychotics	Death (n = 1 (6%)), not CBD-related), headache (n = 1 (6%)) in the CBD group	Kock *et al.,* Nov 2021 [19]
RCT, double-blind, placebo-controlled, multicenter trial	Age 18-65 (mean of 36.7 [10.5]); 80 subjects with a treatment-refractory social anxiety disorder or panic disorder with agoraphobia (39 CBD group; 41 placebo)	300 mg; oral	8 weeks, once a week, 2 h before therapist-assisted outpatient sessions	Not reported	CBD group 4/39: 1 isolated dizziness; 1 isolated drowsiness; 1 recurrent tiredness; 1 recurrent feeling of a strong blood flow placebo group 6/41: 1 isolated sweating, hot flushes, nausea, blurred vision, and a bad taste in the mouth; 1 isolated flu and gout attack; 1 isolated suicidal thoughts; 1 recurrent tiredness; 1 recurrent drowsiness; 1 recurrent headache	Kwee *et al.,* 2022 [58]
Open-label trial	Age (mean 37.8 [7.8]); 5 subjects, who were taking buprenorphine for an average of 37.8 months and had psychiatric comorbidities	600 mg; oral	3 days	Buprenorphine	No significant AEs related to add-on CBD treatment apart from moderate sedation (n = 1)	Suzuki *et al.,* 2020 [64]
Open-label, multi-site trial	Age 6-17; 20 subjects with X-fragile Syndrome	50-250 mg/day; transdermal	Twice daily, 12 weeks	Valproate, lamotrigine	AEs mild/moderate in 17/20 (85%); 6/20 (30%) with at least 1 AE possibly/probably CBD-related, including 2 patients with application site disorders (mild dryness, moderate rash): gastroenteritis (n = 5 (25%)), vomiting (n = 2 (10%)), upper respiratory tract infection (n = 2 (10%))	Heussler *et al.,* 2019 [54]
Open-label trial	Age 2-18; 3 subjects with SYNGAP1 mutation.	10-23 mg/kg/day as add-on therapy; oral	11-14 months	Valproate, lamotrigine, clobazam, zonisamide	Not reported	Kuchenbuch *et al.,* 2020 [55]
**Pain Management and Inflammatory Response**						
RCT, double-blind, placebo-controlled trial	Age 18-75; 99 opioid-naive subjects undergoing Arthroscopic Rotator Cuff Repair to assess CBD analgesic effect: 47 placebo group, 54 CBD group	25 or 50 mg (three times a day); oral	14 days	Opioid	AEs in 4/54 in the CBD group and 4/47 in the placebo group (increased transaminase level); no differences between CBD and placebo groups	Alaia *et al.,* 2022 [80]
RCT, double-blind, placebo-controlled trial	Age 21-50 (mean 32 [8]); 17 healthy non-cannabis-using subjects receiving 0-200-400-800 mg CBD in a randomized order	200, 400, or 800 mg single dose; oral	Single dose	Not reported	AEs mild/moderate; no differences between placebo and CBD groups: Lethargy (n = 5 (29%)), upset stomach including gas and cramps (n = 1 (6%)), subtle mood change (n = 1 (6%)) in the placebo group; lethargy (n = 5 (29%)), upset stomach including gas and cramps (n = 1 (6%)) in the CBD 200 mg group; lethargy (n = 5 (29%)), upset stomach including gas and cramps (n = 4 (24%)), frequent urination (n = 1 (6%)), wooziness (n = 1 (6%)) in the CBD 400 mg group; lethargy (n = 5 (29%)), upset stomach including gas and cramps (n = 4 (24%)), wooziness (n = 1 (6%)) in the CBD 800 mg group	Arout *et al.,* 2021 [22]
Open-label, single-center trial	Age mean 64.2 (11.0); 18 subjects with symptomatic thumb basal joint arthritis	2 mL; transdermal	2 weeks	Not reported	No significant AEs related to CBD	Heinemann *et al.,* 2022 [82]
RCT, double-blinded, placebo-controlled trial	Age (mean 62.0 [56.25-68] CBD group; mean 61.5 [53-70.75] placebo group); 136 subjects with hand osteoarthritis or psoriatic arthritis: 70 CBD group; 66 placebo group.	20, 30 mg; oral	12 weeks	Paracetamol, NSAIDs, antiepileptics, codeine, tramadol, opioids	4 SAEs reported; Placebo Group SAEs: acute shoulder fracture, malignant hypertension; CBD Group: ductal carcinoma, lipothymia	Vela *et al.,* 2022 [81]
RCT, double-blind, placebo-controlled trial	Age (mean 37.8 [11]); 42 subjects with COVID-19 on a placebo Age (mean 40.9 [10.9]); 49 subjects with COVID-19 on CBD	300 mg; oral	2 weeks	Paracetamol, acetaminophen, dipyrone	AEs mild/moderate: somnolence (n = 38 (78%)), fatigue (n = 38 (78%)), decreased appetite (n = 38 (78%)), lethargy (n = 25 (51%)), weight loss (n = 24 (49%)), nausea (n = 23 (47%)), diarrhea (n = 21 (43%)), increased appetite (n = 17 (35%)), fever (n = 11 (22%)), weight gain (n = 10 (20%)), vomiting (n = 6 (12%)), headache (n = 4 (8%)), abdominal pain (n = 4 (8%)), rash (n = 3 (6%)), bitter mouth (n = 3 (6%)) in 49/49 in CBD group; somnolence (n = 33 (79%)), fatigue (n = 33 (79%)), decreased appetite (n = 32 (76%)), lethargy (n = 15 (36%)), weight loss (n = 22 (52%)), nausea (n = 16 (38%)), diarrhea (n = 20 (48%)), increased appetite (n = 10 (24%)), fever (n = 15 (46%)), weight gain (n = 8 (19%)), vomiting (n = 4 (10%)), headache (n = 3 (7%)), abdominal pain (n = 2 (5%)), bitter mouth (n = 4 (10%)) in 42/42 in placebo group	Crippa *et al.,* 2021 [62]

**Abbreviations:** AD, Alzheimer’s disease; AEs, adverse events; AEDs, antiepileptic drugs; CBD, cannabidiol; CYP, cytochrome P450; DEE, Developmental and Epileptic Encephalopathy; DDI, drug-drug interaction; DS, Dravet syndrome; ES, epileptic spasm; LGS, Lennox-Gastaut syndrome; PD, Parkinson’s disease; OLE, open-label extension; RBD, REM behavior disorder; RCT, randomized controlled trial; THC, Δ9-tetrahydrocannabinol ; TRE, treatment-resistant epilepsy; TSC, Tuberous Sclerosis Complex.

## LIST OF ABBREVIATIONS

AD: Alzheimer’s Disease
AEs: Adverse Events
AEDs: Antiepileptic Drugs
CBD: Cannabidiol
CYP: Cytochrome P450
DEE: Developmental and Epileptic Encephalopathy
DDI: Drug-drug Interaction
DS: Dravet Syndrome
EMA: European Medicine Agency
ES: Epileptic Spasm
FDA: US Food and Drug Administration
LGS: Lennox-gastaut Syndrome
PD: Parkinson’s Disease
OLE: Open-label Extension
OTC: Over-the-counter
PPAR-γ: Peroxisome Proliferator-activated Receptor-gamma
RBD: REM Behaviour Disorder
RCT: Randomized Controlled Trial
THC: Δ9-Tetrahydrocannabinol
TRE: Treatment-resistant Epilepsy
TSC: Tuberous Sclerosis Complex
UGT: 5'-diphosphoglucuronosyltransferase

## SUPPLEMENTARY MATERIAL

PRISMA checklist is available as supplementary material on the publisher’s website along with the published article.
